# Identification of microRNAs associated with the aggressiveness of prolactin pituitary tumors using bioinformatic analysis

**DOI:** 10.3892/or.2021.8081

**Published:** 2021-05-17

**Authors:** Zihao Wang, Lu Gao, Xiaopeng Guo, Chenzhe Feng, Kan Deng, Wei Lian, Bing Xing

Oncol Rep 42: 533-548, 2019; DOI: /10.3892/or.2019.7173

Following the publication of this article, an interested reader contacted the authors about some possible anomalies in the presentation of the data in Table I, and they have realized that this Table contained some errors. Two different entries for the miRNA hsa-miR-886-3p were inadvertently included in the Table, due to there being two different microarray IDs for this miRNA when performing differential analysis by GEO2R (Owing to an oversight, the repeated miRNA was not deleted; therefore, the row of data for the second has-miR-886-3p entry, comprising P-value 0.00235, t-value, 3.82, B-value, −4.58 and logFC, 3.2 has been deleted, and the correct data row for the miRNA hsa-miR-513a-5p has been inserted.) A corrected version of the Table is shown opposite (the corrected data row entry is highlighted in bold).

The authors sincerely apologize for the errors that were introduced during the preparation of this Table, and thank the reader of their article who drew this matter to their attention. Furthermore, they regret any inconvenience that this mistake has caused.

## Figures and Tables

**Table I.** Top 10 upregulated and downregulated DEMs between aggressive and nonaggressive prolactin pituitary tumors.

miRNAs (DEMs)	P-value	t	B	logFC
Upregulated				
hsa-miR-489	0.00677	3.25	−4.58	7.07
hsa-let-7d*	0.02591	2.53	−4.58	6.09
hsa-miR-138-1*	0.02569	2.54	−4.58	5.26
hsa-miR-886-3p	0.00191	3.94	−4.58	4.36
hsa-miR-576-5p	0.04773	2.2	−4.59	3.83
hsa-miR-135b	0.01671	2.77	−4.58	3.72
hsa-miR-137	0.03877	2.32	−4.59	3.29
**hsa-miR-513a-5p**	**0.02279**	**2.60**	**−4.58**	**2.94**
hsa-miR-551b	0.02074	2.66	−4.58	3.04
hsa-miR-296-3p	0.04524	2.23	−4.59	3.02
Downregulated				
hsa-miR-520b	0.00732	−3.21	−4.58	−6.36
hsa-miR-875-5p	0.04037	−2.29	−4.59	−5.66
hsa-miR-671-3p	0.01453	−2.85	−4.58	−5.49
hsa-miR-372	0.00348	−3.61	−4.58	−5.49
hsa-miR-586	0.02631	−2.53	−4.58	−5.44
hsa-miR-367*	0.02421	−2.57	−4.58	−4.84
hsa-miR-302b	0.01052	−3.02	−4.58	−4.49
hsa-miR-187	0.0322	−2.42	−4.59	−4.35
hsa-miR-193b*	0.02207	−2.62	−4.58	−4.31
hsa-miR-452*	0.00322	−3.65	−4.58	−4.17

miRNA names with ‘*’ are also mature miRNAs as annotated in miRBase (http://www.mirbase.org). For example, hsa-let-7d* is hsa-let-7d-3p; hsa-miR-138-1* is hsa-miR-138-1-3p; hsa-miR-367* is hsa-miR-367-5p; hsa-miR-193b* is hsa-miR-193b-5p; hsa-miR-452* is hsa-miR-452-3p. DEMs, differentially expressed miRNAs; hsa, *Homo sapiens*.

